# A 5:2 Intermittent Fasting Regimen Ameliorates High‐Fat Diet‐Induced MASLD‐Associated Skeletal Muscle Impairment in Mice

**DOI:** 10.1002/jcsm.70398

**Published:** 2026-10-04

**Authors:** Ruohua Wang, Rui Yang, Dan Hu, Tonghe Xin, ZiXin Yang, Yue Guan, Yucun Niu

**Affiliations:** ^1^ Department of Nutrition and Food Hygiene, Public Health College Harbin Medical University Harbin China

**Keywords:** 5:2 intermittent fasting, gut microbiota, intestinal barrier, metabolic dysfunction‐associated steatotic liver disease (MASLD), skeletal muscle quality

## Abstract

**Background:**

Metabolic dysfunction–associated steatotic liver disease (MASLD) is a highly prevalent chronic liver disorder worldwide. Its pathogenesis is closely linked to high‐fat diets (HFDs) and is frequently accompanied by decreased skeletal muscle quality. Although intermittent fasting (IF) has been shown to effectively alleviate hepatic steatosis and inflammation, its impact on skeletal muscle quality remains unclear.

**Methods:**

Eight‐week‐old male C57BL/6J mice were randomly divided into two groups and fed a normal chow (NC) or 45% HFD for 14 weeks to induce MASLD. Subsequently, the MASLD model mice were subjected to a 5:2 intermittent fasting (IF5:2) regimen for 12 cycles (2 days of fasting per week, with ad libitum HFD feeding on the remaining 5 days). Grip strength, liver weight and quadriceps muscle mass were measured at sacrifice. Serum samples were collected for biochemical analysis and lipopolysaccharide (LPS) measurement. Liver, skeletal muscle and intestinal tissues were harvested for histopathological examination. Faecal samples were analysed by metagenomic sequencing. The mRNA expression of bile acid receptors and inflammatory genes was detected by qRT‐PCR. Targeted free fatty acid profiling was performed in liver and skeletal muscle tissues.

**Results:**

Compared with the continuous HFD group, the IF5:2 group showed no significant difference in cumulative energy intake but exhibited significantly reduced body weight, as well as alleviated hepatic steatosis and inflammation. IF5:2 significantly increased grip strength and skeletal muscle cross‐sectional area and markedly downregulated mRNA expression of inflammation‐related genes in muscle tissue. Regarding intestinal barrier function, the IF5:2 group displayed significantly higher mRNA expression of intestinal tight junction–related genes, a thicker colonic wall and lower serum LPS levels compared with the HFD group. Metagenomic analysis suggested that IF5:2 effectively remodelled HFD‐induced gut microbiota dysbiosis and significantly enriched beneficial bacteria including *Limosilactobacillus reuteri* and *Faecalibaculum rodentium*, accompanied by significant upregulation of microbial secondary bile acid biosynthesis pathways. mRNA expression levels of bile acid receptors and their downstream regulatory genes in the intestine, liver and skeletal muscle were significantly elevated in the IF5:2 group. IF5:2 treatment significantly reduced free fatty acid levels in the liver and skeletal muscle, which were negatively correlated with the gene expression of bile acid‐related receptors in these two tissues.

**Conclusions:**

Cyclic IF5:2 effectively ameliorates HFD‐induced MASLD and protects against skeletal muscle impairment. It represents a promising dietary intervention with dual beneficial effects on alleviating hepatic steatosis and maintaining skeletal muscle quality.

## 1 Introduction

Metabolic dysfunction–associated steatotic liver disease (MASLD) is the most prevalent chronic liver disease worldwide. Its pathogenesis is closely associated with high‐fat diets (HFDs), gut microbiota dysbiosis and metabolic imbalance, making it a major public health threat to human health [1]. Sustained hepatic metabolic disturbances can further impair skeletal muscle health and accelerate muscle atrophy through inflammation‐mediated pathways and abnormal metabolite production [2]. A growing body of evidence indicates that MASLD is accompanied by reduced skeletal muscle mass [3]. Therefore, the development of non‐pharmacological interventions that can both improve MASLD and protect skeletal muscle against impairment is of great clinical significance and research value.

Intermittent fasting (IF) has gained extensive attention as a dietary strategy to improve metabolic health. Currently, IF is mainly classified into three categories based on fasting patterns: alternate‐day fasting, whole‐day fasting and time‐restricted feeding (TRF). The widely studied 5:2 intermittent fasting (IF5:2) regimen—two nonconsecutive days of fasting or very‐low‐calorie intake per week and ad libitum feeding on the remaining 5 days—belongs to the whole‐day fasting category [4]. Recent rodent and clinical studies of IF5:2 have demonstrated its beneficial effects on reducing body weight, alleviating hepatic steatosis and attenuating inflammation [5, 6]. Although human studies have shown that continuous prolonged fasting does not alter muscle metabolism or performance, nor does it induce structural or inflammatory changes [7], other studies have suggested that the combination of minimal food intake and prolonged fasting in IF regimens may be unfavourable for muscle protein metabolism and exert negative effects on net muscle protein balance [8]. Some studies have reported that IF may reduce lean body mass along with body weight, and although such reduction is not sufficient to cause muscle atrophy, its impact on skeletal muscle mass warrants attention [9]. Notably, most clinical IF studies lack comprehensive assessments of skeletal muscle mass, and its direct and definitive impact on muscle mass remains unclear. Studies using TRF in rodents have shown direct beneficial effects on skeletal muscle remodelling [10], but the duration and frequency of TRF differ from those of IF5:2. A murine study using three nonconsecutive 24‐h fasts per week—more similar to IF5:2—showed that IF enhanced hepatic autophagy but did not activate skeletal muscle autophagy, a process critical for maintaining cellular homeostasis and energy balance under stress or fasting conditions [11]. Although this may imply weaker beneficial effects of IF on skeletal muscle than on the liver, other evidence indicates that sufficiently long fasting periods trigger a metabolic switch that activates AMPK in myocytes, which in turn activates SIRT3 and protects myocytes against oxidative stress [12]. Collectively, the specific effects of IF on skeletal muscle quality remain inconsistent and inconclusive in both human and animal studies. As IF becomes increasingly popular in interventions for MASLD, its impact on skeletal muscle quality represents an important issue that must be addressed. However, direct and combined investigations of the effects of IF on both liver and muscle impairment are currently lacking.

Accordingly, we used a mouse model of HFD‐induced MASLD and administered 12 weeks of IF5:2 to investigate its effects on hepatic health and skeletal muscle quality. We aimed to verify whether IF5:2 is a promising dietary intervention that simultaneously improves MASLD and protects skeletal muscle from impairment, thereby providing experimental evidence and theoretical support for its clinical application.

## 2 Materials and Methods

### 2.1 Animal Experimental Design

Seven‐week‐old male C57BL/6J mice were purchased from Beijing Vital River Laboratory Animal Technology Co. Ltd. and acclimated for 1 week. Mice were randomly divided into two groups and fed a normal chow (NC) or 45% HFD for 14 weeks to induce MASLD (detailed diet composition is shown in Table S1). At the end of the 14‐week induction period, five mice were randomly selected from the NC group and the HFD‐fed group, respectively, and euthanised for liver histopathological examination to confirm successful establishment of the MASLD model. The remaining HFD‐fed mice were then randomised by body weight into two groups: a continuous HFD group and a IF5:2 group, followed by a 12‐week intervention (Figure 1a). The IF5:2 regimen was performed as previously reported [5]: two nonconsecutive days of fasting per week with free access to water and ad libitum HFD feeding on the other 5 days. Mice were housed in a specific pathogen‐free (SPF) facility with controlled temperature (21°C ± 2°C), humidity (50% ± 5%) and a 12‐h light/dark cycle. All mice were sacrificed in the fed state. Mice in the IF5:2 group were sacrificed 48 h after completion of the last fasting cycle. This ensured that IF5:2 mice were in a fed state at sacrifice, consistent with ad libitum‐fed control mice. All animal procedures were approved by the Ethics Committee of Harbin Medical University (HMUIRB2026029).

**FIGURE 1:**
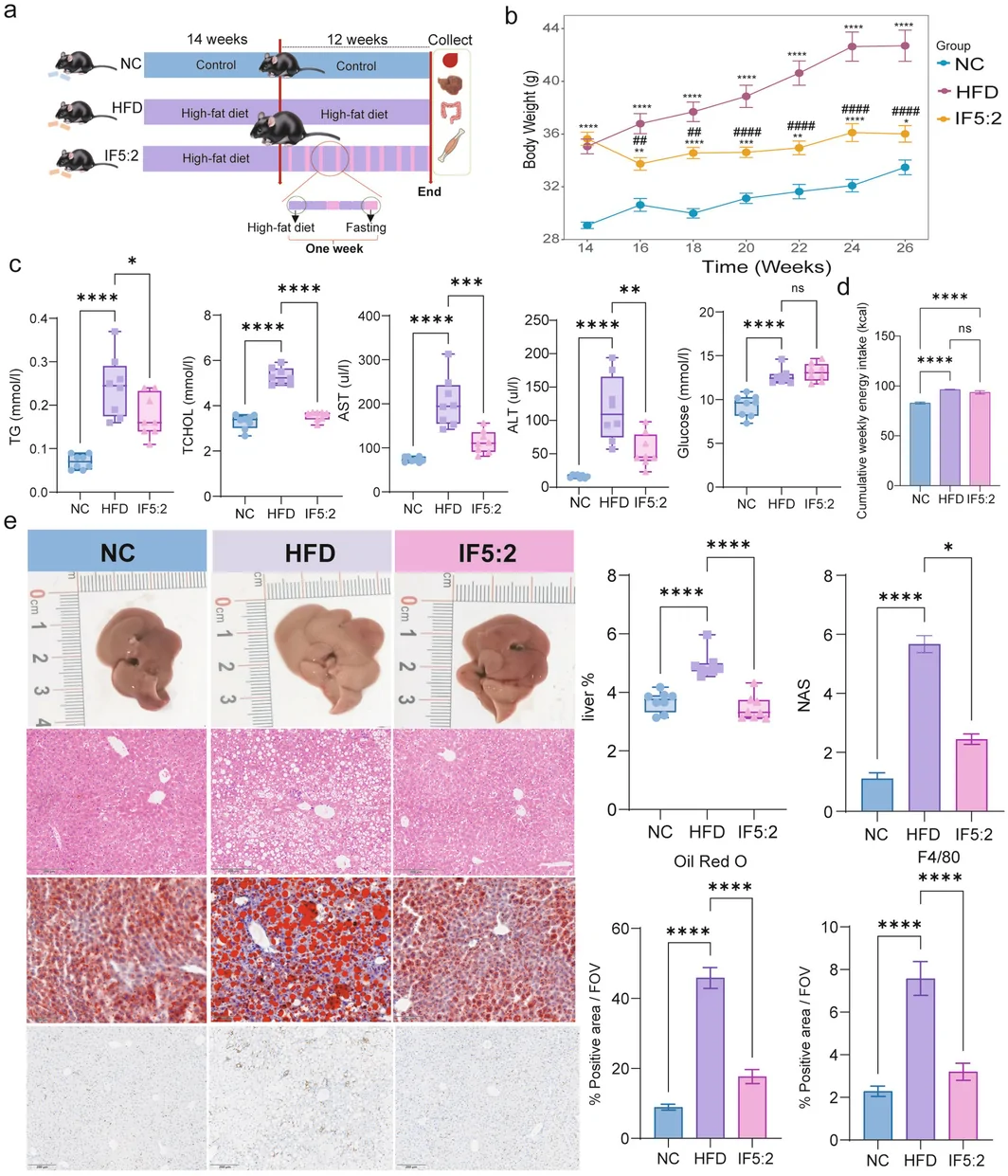
Experimental design and beneficial effects of 5:2 intermittent fasting (IF5:2) on metabolic dysfunction–associated steatotic liver disease (MASLD). (a) Schematic of the experimental protocol. (b) Body weight changes in mice during IF5:2 intervention. (c) Serum biochemical parameters in mice after IF5:2 intervention (*n* = 8). (d) Cumulative food intake per 2 weeks in each group. (e) Liver morphology and histopathological analysis: liver‐to‐body weight ratio, NAS histopathological score, Oil Red O‐positive lipid droplet area percentage and F4/80‐positive area percentage (three fields per section, three sections per group). * indicates comparisons with the NC group, and # indicates comparisons with the HFD group. *, #, *p* < 0.05; **, ##, *p* < 0.01; ***, ###, *p* < 0.001; ****, ####, *p* < 0.0001.

### 2.2 Measurement of Mouse Grip Strength

Skeletal muscle contractile function was assessed using a YLS‐13A grip strength meter. Mice were acclimated to a quiet environment for 10 min before testing. During measurement, each mouse was gently pulled backward horizontally until it released the grid, and the maximum grip force (g) was recorded. Each mouse underwent five trials at 1‐min intervals, and the maximum grip force value was recorded. All data were normalised to body weight for subsequent statistical analysis. Excessive pulling was avoided to prevent injury, and consistent operation was maintained.

### 2.3 Serum Biochemical Parameters

Serum levels of glucose, total triglycerides (TG), aspartate aminotransferase (AST) and alanine aminotransferase (ALT) were measured using a ROCHE Modular P800 automatic biochemical analyser. Serum levels of tumour necrosis factor‐α (TNF‐α), interleukin‐6 (IL‐6) (Lupin Biotechnology, Shanghai, China) and lipopolysaccharide (LPS) (Mianfei Biotechnology, Jiangsu, China) were determined using enzyme‐linked immunosorbent assay (ELISA) kits.

### 2.4 Histopathological Staining of Liver, Intestine and Skeletal Muscle

The left liver lobe, colon and quadriceps femoris were fixed, dehydrated, embedded in paraffin and stained with haematoxylin and eosin (H&E). Paraffin‐embedded liver sections were processed for immunohistochemistry with anti‐F4/80 antibody (detailed procedures are described in Supporting Information S1.1–S1.2). Paraformaldehyde‐fixed liver tissues were cryosectioned and stained using an Oil Red O staining kit (Solarbio, China) following the manufacturer's protocol. Cross‐sectional area (CSA) of quadriceps myofibres, F4/80‐positive area and lipid droplet area were quantified using the ImageJ software.

### 2.5 Faecal Metagenomic Sequencing and Analysis

During the post‐intervention refeeding phase, faeces were collected after perianal cleaning with 75% ethanol. Genomic DNA was extracted using the FastPure Stool DNA Isolation Kit (Magnetic bead; MJYH, Shanghai, China). DNA concentration, purity and integrity were assessed. DNA was fragmented to ~350 bp, and metagenomic sequencing was performed on an Illumina NovaSeq X Plus platform. Raw reads were aligned to the host genome to remove contaminants. Clean reads were assembled, and open reading frames (ORFs) were predicted and translated into amino acids. A nonredundant gene catalogue was constructed by clustering genes at 90% identity and 90% coverage. Gene abundance was quantified by aligning high‐quality reads to the catalogue at 95% identity. Taxonomic and functional annotations were derived from the NR and KEGG databases, respectively.

### 2.6 Quantitative Real‐Time PCR Analysis

Total RNA was extracted from approximately 50 mg of liver, skeletal muscle and ileum tissues using TRIzol reagent (Vazyme, Nanjing, China). RNA was reverse transcribed into cDNA using a high‐capacity cDNA reverse transcription kit (Vazyme, Nanjing, China). qRT‐PCR was performed using SYBR Green PCR Master Mix on a Roche LightCycler 480 real‐time PCR system. Gene‐specific primers were synthesised by Sangon Biotech Co. Ltd. (Jiangsu, China); primer sequences are listed in Table 1. Relative gene expression was calculated using the 2−ΔΔCt method.

**TABLE 1:** Real‐time quantification of PCR primers.

Gene	Forward primer sequence	Reverse primer sequence
*Fxr*	CCCACTGGCATGCCCTTTT	TCTGGAAATGACCTGCCCTC
*Cyp7a1*	GGGCAGGCTTGGGAATTTTG	ACAGCTACTAGGGGGCTTCA
*Ppara*	CGGGAAAGACCAGCAACAAC	ATAGCAGCCACAAACAGGGA
*Cpt1a*	TCAAGCCAGACGAAGAACATC	TGGTAGGAGAGCAGCACCTT
*Acox1*	GGAATTTGGCATCGCAGACC	GGACGGGTGCATCCATTTCT
*Foxo3*	TGAAGGGAAGGAGCCGAGGTA	CGACTCTGTGGCTCGAACTC
*Fgfr4*	GAAGGTGGTCAGTGGGAAGTC	TTCTTCTCCATGCGTTGGGG
*Klb*	GACACAACCTGATCAAGGCAC	CCTTCTGATGAGGGCGGAAG
*Tgr5*	CCTGGCAAGCCTCATCGTC	AGCAGCCCGGCTAGTAGTAG
*Pkaca*	CACCCTATCACTCCCTGGCT	AACCTTCCAATCCGCCGTAG
*Trim63*	GAGGGGCTACCTTCCTCTCA	TTTACCCTCTGTGGTCACGC
*Fbxo32*	GCCCTCCACACTAGTTGACC	GACGGATTGACAGCCAGGAA
*Muc2*	TCCTGACCAAGAGCGAACAC	ACAGCACGACAGTCTTCAGG
*Cldn4*	TGATTATGGTGCCCGTGTCC	CGAGTAGGGCTTGTCGTTGC
*Ocln*	CCCAGGCTTCTGGATCTATGT	TCCATCTTTCTTCGGGTTTTCA
*Tjp1*	CGTTCCGGGGAAGTTACGTG	TGGAGGTTTCCCCACTCTGA
*Il6*	GAAATGATGGATGCTACCAAACTG	GACTCTGGCTTTGTCTTTCTTGTT
*Tnf*	CCTGTAGCCCACGTCGTAG	GGGAGTAGACAAGGTACAACCC
*Gapdh*	AGGAGCGAGACCCCACTAACA	AGGGGGGCTAAGCAGTTGGT

### 2.7 Targeted Free Fatty Acid (FFA) Profiling in Liver and Skeletal Muscle

Approximately 50 mg of liver and 100 mg of skeletal muscle were homogenised in ice‐cold PBS. The upper organic phase was extracted, evaporated to dryness under nitrogen and methylated. Samples were stored at 4°C until analysis by gas chromatography–mass spectrometry (GC–MS; Thermo Finnigan, Austin, TX, United States) (detailed procedures are described in Supporting Information S1.3) [13].

### 2.8 Statistical Analysis

Quantitative data are expressed as mean ± standard error of the mean (mean ± SEM) unless otherwise stated. For continuous variables, if not specified otherwise, the standard approach was to use one‐way ANOVA followed by Tukey's post hoc test for statistical analysis. All statistical analyses were performed using GraphPad Prism 9.0 or R 4.4.1. All tests were two‐sided, and *p* < 0.05 was considered statistically significant.

## 3 Results

### 3.1 IF5:2 Ameliorates HFD‐Induced MASLD in Mice

After 14 weeks of HFD feeding, body weight was significantly higher in HFD‐fed mice than in the NC group, accompanied by marked elevations in serum glucose, lipid profiles, ALT and AST. Hepatic histopathology confirmed successful establishment of the MASLD model (Figure S1a–i). Following 12 weeks of IF5:2 intervention, body weight was significantly reduced in the IF5:2 group relative to the HFD group (Figure 1b). Serum TG, TC, ALT and AST were also markedly decreased (Figure 1c). Notably, no significant difference in blood glucose was observed between IF5:2 and HFD mice (Figure 1c). As blood glucose was assessed in the post‐fasting refeeding phase, this finding may be attributable to compensatory hyperphagia in IF5:2 animals. HFD mice had greater daily total and fat‐derived energy intake relative to NC mice (Figure S2a–d). IF5:2 mice displayed prominent compensatory hyperphagia during refeeding, accompanied by significantly higher daily energy and fat intake than HFD mice (Figure S2a–d). However, cumulative weekly food mass, total energy intake and fat‐derived energy intake were similar between the IF5:2 and HFD groups (Figure 1d; Figure S2e–g), suggesting that elevated food intake upon refeeding offsets the energy deficit during fasting. Consistent with reduced body weight and improved systemic metabolism, IF5:2 intervention significantly lowered the liver‐to‐body weight ratio compared with the HFD group. In contrast to the yellow, steatotic appearance of the livers in HFD mice, those from IF5:2 mice exhibited a healthier gross morphology. Hepatic histopathology further confirmed that IF5:2 effectively alleviated hepatic lipid accumulation and inflammation (Figure 1e; Figure S3a–d). In addition, the weights of omental, subcutaneous, perirenal and epididymal adipose tissues were markedly decreased in the IF5:2 group relative to the HFD group, whereas no significant difference was observed in brown adipose tissue weight (Figure S3e–j). Collectively, these findings demonstrate that IF5:2 intervention effectively prevented the progression of HFD‐induced MASLD and improved hepatic steatosis and metabolic health.

### 3.2 IF5:2 Attenuates HFD‐Induced Skeletal Muscle impairment and Inflammation in MASLD Mice

Histopathological analysis revealed that the CSA of skeletal muscle fibres was markedly decreased in HFD‐fed mice after 26 weeks compared with the NC group, verifying that long‐term HFD leads to muscle impairment in MASLD mice (Figure 2a–e). Following IF5:2 intervention, although the absolute quadriceps mass in mice was not significantly higher than that in the HFD group, the muscle‐to‐body weight ratio and grip strength normalised to body weight were markedly increased relative to the HFD group and were comparable with those of the NC group (Figure 2a,b; Figure S4a,b). To further characterise the association between body weight and quadriceps mass, we performed linear regression analysis of these two variables within each group. A significant positive correlation was only observed in the HFD group, indicating that the magnitude of body weight gain substantially exceeded the increase in quadriceps mass (Figure S4c). Moreover, mRNA expression levels of the inflammatory cytokines *Il6* and *Tnf* in skeletal muscle were significantly lower in the IF5:2 group than in the HFD group (Figure 2c,d). Meanwhile, skeletal muscle fibre CSA was significantly larger in the IF5:2 group than in the HFD group, further confirming the protective effect of IF5:2 against HFD‐induced muscle impairment (Figure 2e). These results indicate that IF5:2 intervention not only improves MASLD but also effectively mitigates HFD‐induced skeletal muscle impairment.

**FIGURE 2:**
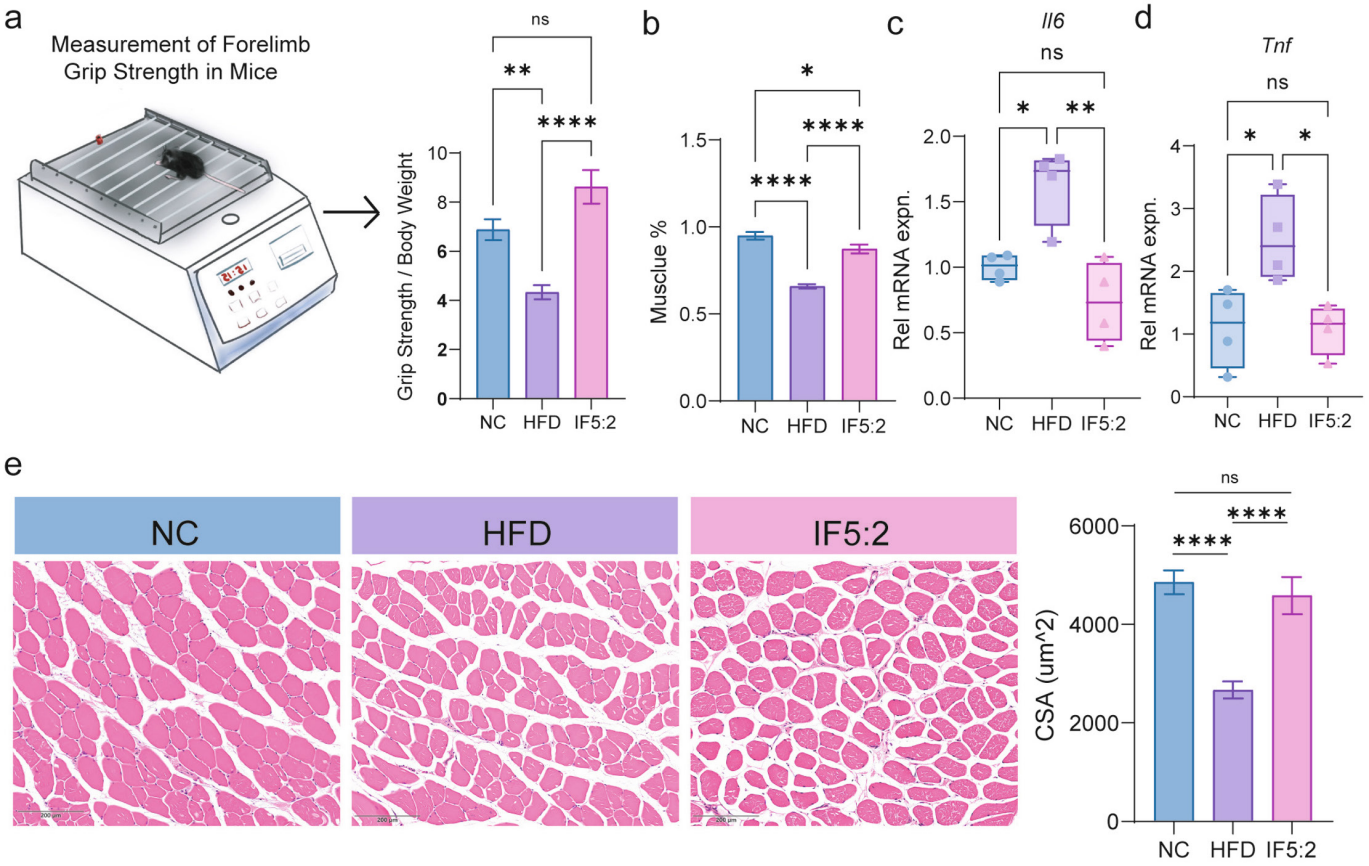
Analysis of skeletal muscle grip strength, inflammatory mRNA expression and HE staining‐based cross‐sectional area in mice. (a) Grip strength measurement of mice (*n* = 8). (b) Quadriceps muscle mass normalized to body weight (*n* = 8). (c–d) Quantitative real‐time PCR (qRT‐PCR) analysis of inflammatory factors *Il6* and *Tnf* in skeletal muscle (*n* = 4). (e) Haematoxylin–eosin (HE) staining of skeletal muscle and statistical analysis of cross‐sectional area (CSA). *p < 0.05, **p < 0.01, ***p < 0.001, and ****p < 0.0001.

### 3.3 IF5:2 Enhances Intestinal Barrier Function and Reduces Intestinal Permeability

An intact intestinal barrier serves as a critical defence against the invasion of harmful luminal substances. Accumulating evidence indicates that long‐term HFD impairs intestinal barrier integrity and reduces intestinal wall thickness [14]. In our study, intestinal histopathology showed that intestinal wall thickness (marked by blue short lines) was significantly decreased in the HFD group compared with the NC group, accompanied by obvious inflammatory cell infiltration (marked by red circles) (Figure 3a). IF5:2 intervention significantly increased intestinal wall thickness and alleviated inflammatory cell infiltration relative to the HFD group. Further qRT‐PCR analysis revealed that mRNA expression levels of the intestinal barrier marker *Muc2* and tight junction‐related genes *Tjp1*, *Cldn4* and *Ocln* were significantly downregulated in the HFD group but markedly upregulated in the IF5:2 group (Figure 3b–e). Serum LPS measurement revealed that LPS levels were significantly higher in the HFD group than in both the NC and IF5:2 groups, with no significant difference between the IF5:2 and NC groups (Figure 3f). These results indicate that IF5:2 effectively alleviates HFD‐induced intestinal barrier damage and enhances intestinal integrity.

**FIGURE 3:**
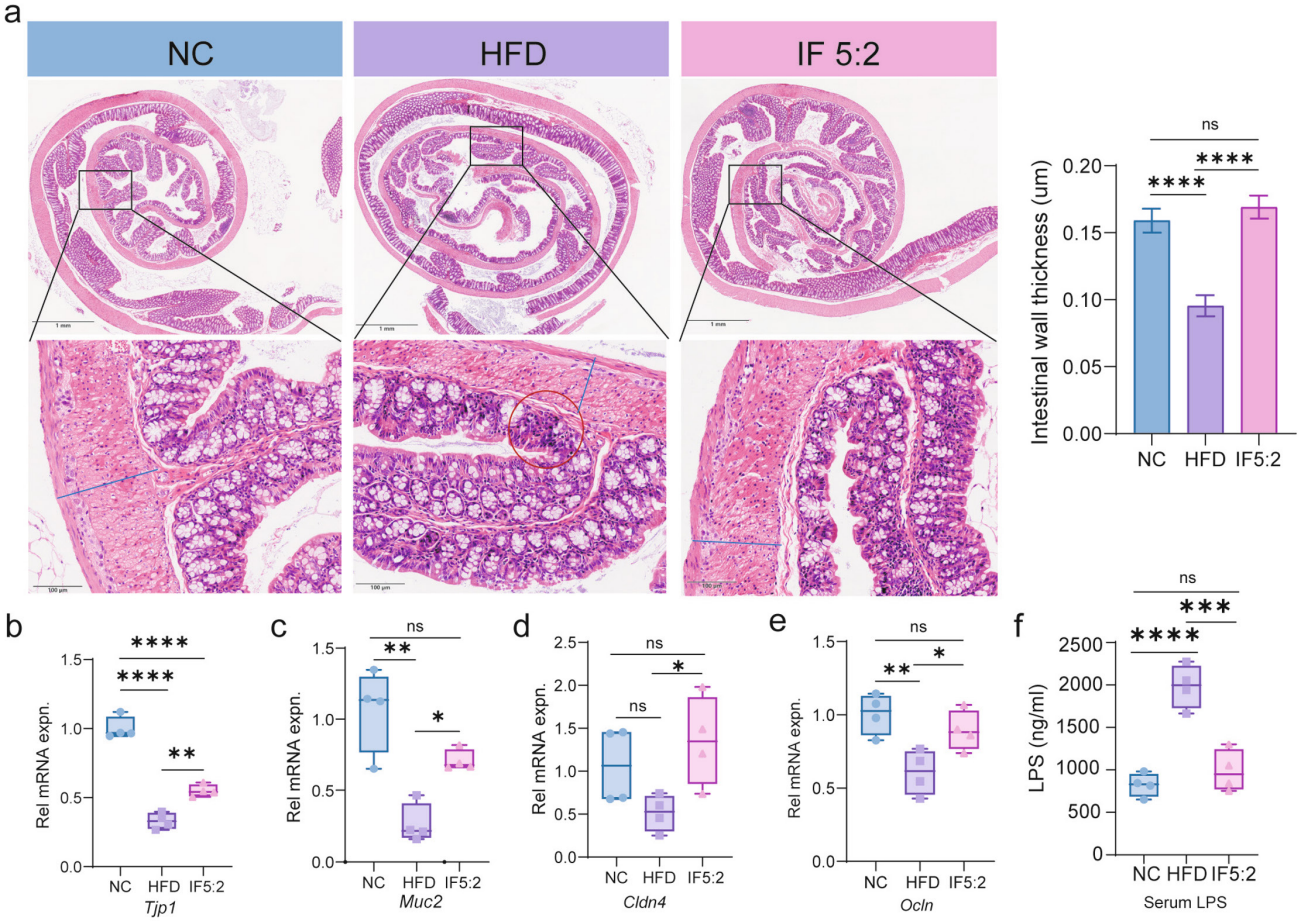
HE staining of colon and expression of intestinal barrier–related genes. (a) Representative HE staining of colon tissues. The blue short lines indicate colon wall thickness, and the red circles indicate inflammatory cell infiltration. (b–e) mRNA expression of intestinal tight junction–related genes (*n* = 4). (f) Serum LPS level (*n* = 4). *p < 0.05, **p < 0.01, ***p < 0.001, and ****p < 0.0001.

### 3.4 IF5:2 May Profoundly Reshape Gut Microbiota Structure and Enrich Bacterial Taxa Possessing Secondary Bile Acid Biosynthetic Potential

To explore the effects of IF5:2 on the composition and function of the gut microbiota in HFD‐fed mice, metagenomic sequencing was performed using faecal samples. Principal coordinate analysis (PCoA) revealed distinct clustering among the NC, HFD and IF5:2 groups, with the IF5:2 group exhibiting a microbiota structure closer to the NC group (Figure 4a), indicating that IF5:2 intervention normalises HFD‐disrupted gut microbiota. Notably, α‐diversity analysis showed no significant differences in microbial richness or evenness among the three groups (Figures S5a–d), suggesting that IF5:2 mainly remodels microbial community structure rather than altering overall diversity. Taxonomic analysis at the species level showed that *Faecalibaculum rodentium* was the dominant species in the NC and IF5:2 groups, whereas *Mucispirillum schaedleri* —a bacterium associated with inflammation—was enriched in the HFD group [15], consistent with the increased serum LPS level in HFD mice (Figure 4b,c). LEfSe analysis identified *Limosilactobacillus reuteri* and *Faecalibaculum rodentium* as characteristic beneficial bacterial species enriched in the IF5:2 group (Figure 4d). The relative abundances of *Faecalibaculum rodentium* and *Limosilactobacillus reuteri* were most significantly increased in the IF5:2 group compared with the HFD group (Figure S5e,f). Functional enrichment analysis of differential KO genes between the HFD and IF5:2 groups showed significant enrichment of the phosphotransferase system (PTS) pathway and the secondary bile acid (SBA) biosynthesis pathway in the IF5:2 group (Figure 4e). In contrast, the LPS biosynthesis pathway was significantly enriched in the HFD group, consistent with the enrichment of pro‐inflammatory bacteria such as *Mucispirillum schaedleri* , indicating increased intestinal endotoxin load and inflammation (Figure 4e). Functional contribution analysis of bacterial taxa involved in the SBA biosynthesis pathway revealed that the differential bacteria taxa between the HFD and IF5:2 groups mainly contributed to bile salt hydrolase (BSH) activity (Figure 4f). Notably, *Faecalibaculum rodentium*, the signature species in the IF5:2 group, exhibited the highest contribution to BSH function (Figure 4f). These results suggest that IF5:2‐mediated gut microbiota remodelling may enhance the genetic potential of the gut microbiota for intestinal bile acid metabolism. Correlation heatmap analysis showed a pronounced bipolar association between gut microbiota and host phenotypic indices. Beneficial bacteria enriched in the IF5:2 group, including *Faecalibaculum rodentium* and *Lactobacillus johnsonii* , were overall positively correlated with muscle strength and negatively correlated with liver function and metabolic parameters (Figure 5a). Conversely, pro‐inflammatory bacteria enriched in the HFD group, such as *Mucispirillum schaedleri* , displayed the opposite correlation pattern (Figure 5a). Although some correlations did not reach statistical significance, this consistent trend suggests a potential association between IF5:2‐mediated gut microbiota remodelling and improved host metabolic health.

**FIGURE 4:**
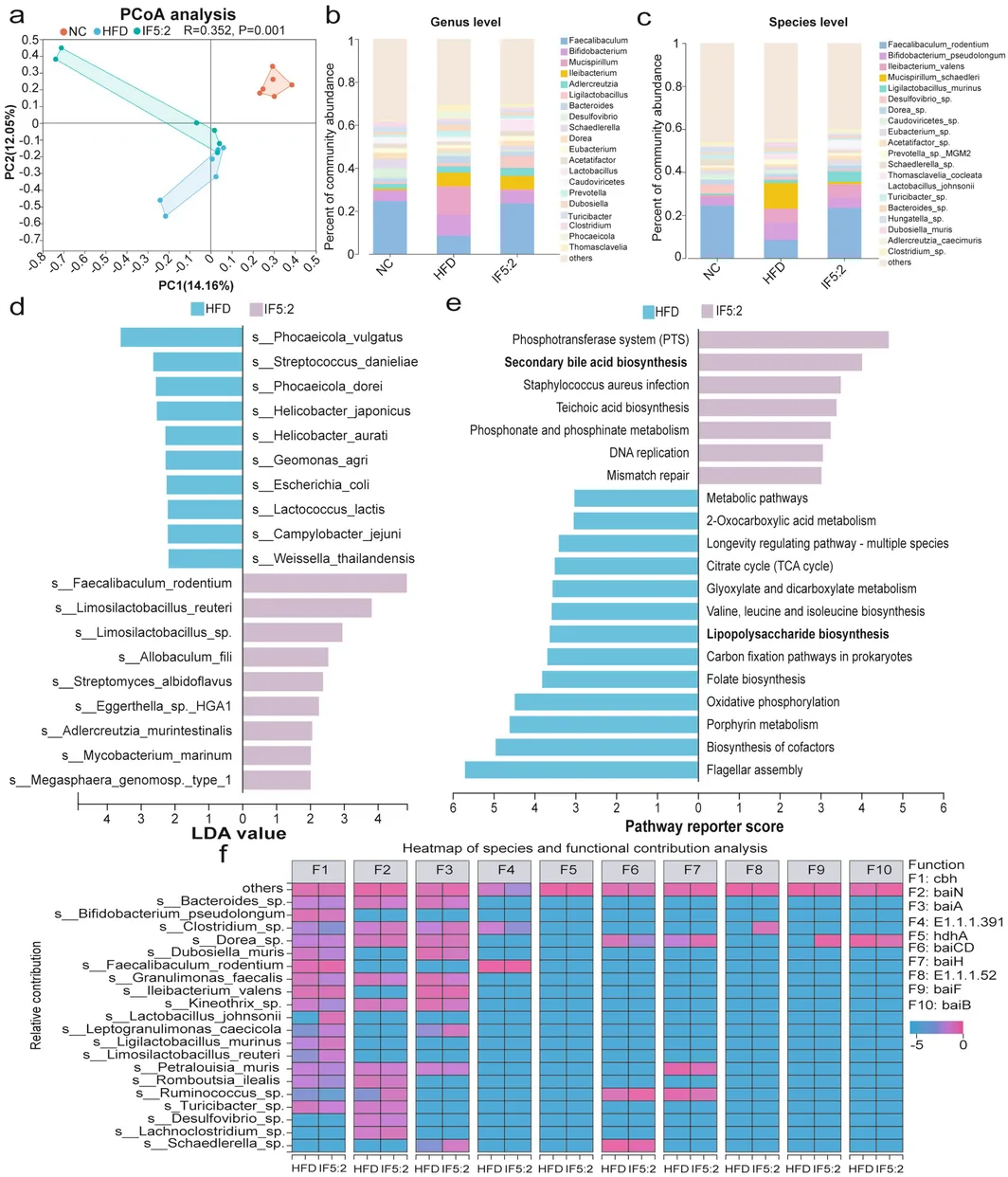
Faecal metagenomic analysis of gut microbiota. (a) Principal coordinate analysis (PCoA) (*n* = 6). (b–c) Relative abundance of gut microbiota at the genus and species levels. (d) Linear discriminant analysis (LDA) effect size (LEfSe) analysis at the species level. Higher LDA score indicates greater enrichment in the corresponding group. (e) KEGG enrichment analysis of differential species between the HFD and IF5:2 groups. (f) Functional contribution analysis of bacterial species from the HFD and IF5:2 groups involved in the secondary bile acid biosynthesis pathway.

**FIGURE 5:**
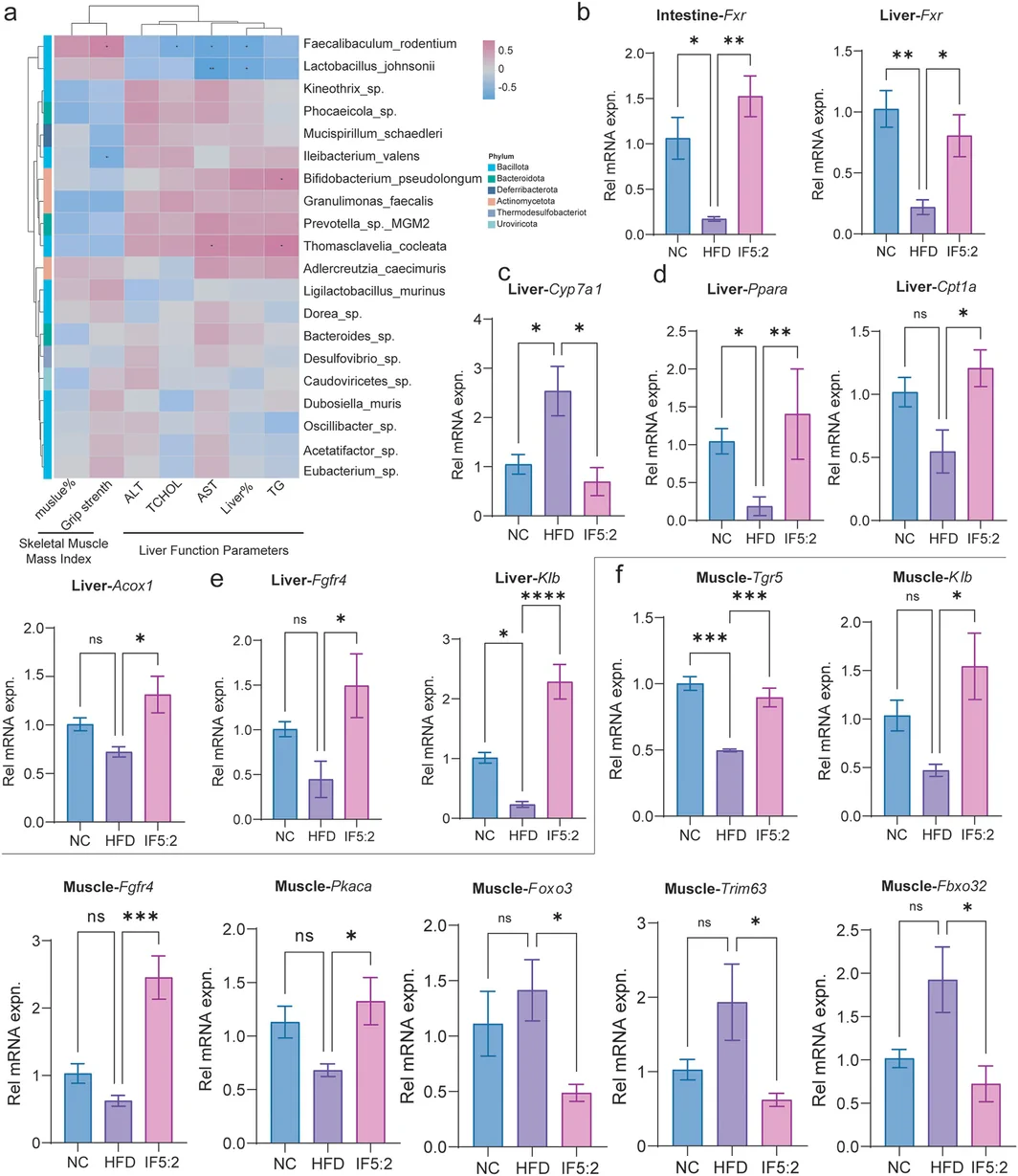
Correlation analysis between bacterial species and skeletal muscle quality or liver function parameters, and mRNA expression of bile acid–related receptors in intestine, liver, and skeletal muscle. (a) Heatmap showing the correlation between differential bacterial species (HFD vs. IF5:2) and skeletal muscle quality or liver function indices. A correlation coefficient < 0 indicates negative correlation, and > 0 indicates positive correlation. (b) mRNA expression of the classic bile acid receptor *Fxr* in intestine and liver. (c–e) mRNA expression of bile acid–related receptor genes in the liver. (f) mRNA expression of bile acid–related receptor genes and muscle atrophy–related genes in skeletal muscle. *p < 0.05, **p < 0.01, ***p < 0.001, and ****p < 0.0001.

### 3.5 IF5:2 Upregulates Bile Acid Receptor Gene Expression in the Intestine, Liver and Skeletal Muscle

Metagenomic analysis revealed that IF5:2 intervention significantly enhanced gut microbiota–mediated SBA biosynthesis. BSH‐active bacteria hydrolyse conjugated bile acids into unconjugated forms, providing substrates for SBA generation. As key signalling molecules, SBAs activate bile acid–related receptors in the intestine, liver and muscle to regulate tissue metabolic homeostasis [16, 17]. In agreement with functional predictions, qRT‐PCR analysis showed that mRNA expression of the canonical bile acid receptor *Fxr* was significantly reduced in the intestine and liver of the HFD group compared with the NC group, but markedly increased in the IF5:2 group relative to the HFD group (Figure 5b). Elevated hepatic *Fxr* further inhibited its downstream target *Cyp7a1*, whose expression was significantly lower in the IF5:2 group than in the HFD group (Figure 5c). Moreover, hepatic expression of *Ppara* and its downstream target genes *Cpt1a* and *Acox1* was significantly upregulated in the IF5:2 group (Figure 5d). Concurrently, mRNA levels of *Fgfr4* (the key receptor for FGF15) and its co‐receptor *Klb* were significantly increased in both liver and muscle of the IF5:2 group compared with the HFD group (Figures 5e,f). Expression of the bile acid–responsive receptor *Tgr5* in skeletal muscle was also significantly higher in the IF5:2 group. Accordingly, *Pkaca* expression was increased, whereas its downstream inhibitory target *Foxo3* and the muscle atrophy–related genes *Trim63* and *Fbxo32* were significantly downregulated in the IF5:2 group (Figure 5f). Collectively, these data reveal that IF5:2 upregulates the expression of key bile acid receptors and downstream genes, suggesting a potential contribution of bile acid signalling to the metabolic benefits of IF5:2 in the liver and muscle.

### 3.6 IF5:2 Significantly Reduces FFA Levels in the Liver and Skeletal Muscle

To investigate the effects of IF5:2 on hepatic and muscle fatty acid metabolism, targeted FFA profiling was performed in liver and skeletal muscle tissues. Orthogonal partial least squares discriminant analysis (OPLS‐DA) showed clear separation between the HFD group and both the NC and IF5:2 groups, with tight intragroup clustering (Figure 6a). Permutation tests confirmed no model overfitting (Q2 < 0), supporting the reliability and generalisability of the models (Figures S6a–d). Total FFA content was significantly lower in both the liver and skeletal muscle of the IF5:2 group than in the HFD group, with no significant difference compared with the NC group (Figure 6b). Heatmap visualisation of FFA composition revealed that C16:0, C18:1, C18:2, C18:3n‐3, C18:4 and C18:3n‐6 were significantly decreased in both the liver and muscle of the IF5:2 group relative to the HFD group (*p* < 0.05, VIP > 1), representing shared differential FFAs between the two tissues (Figure 6b). These findings indicate that IF5:2 intervention effectively reduces lipid accumulation and alleviates metabolic damage in the liver and muscle.

**FIGURE 6:**
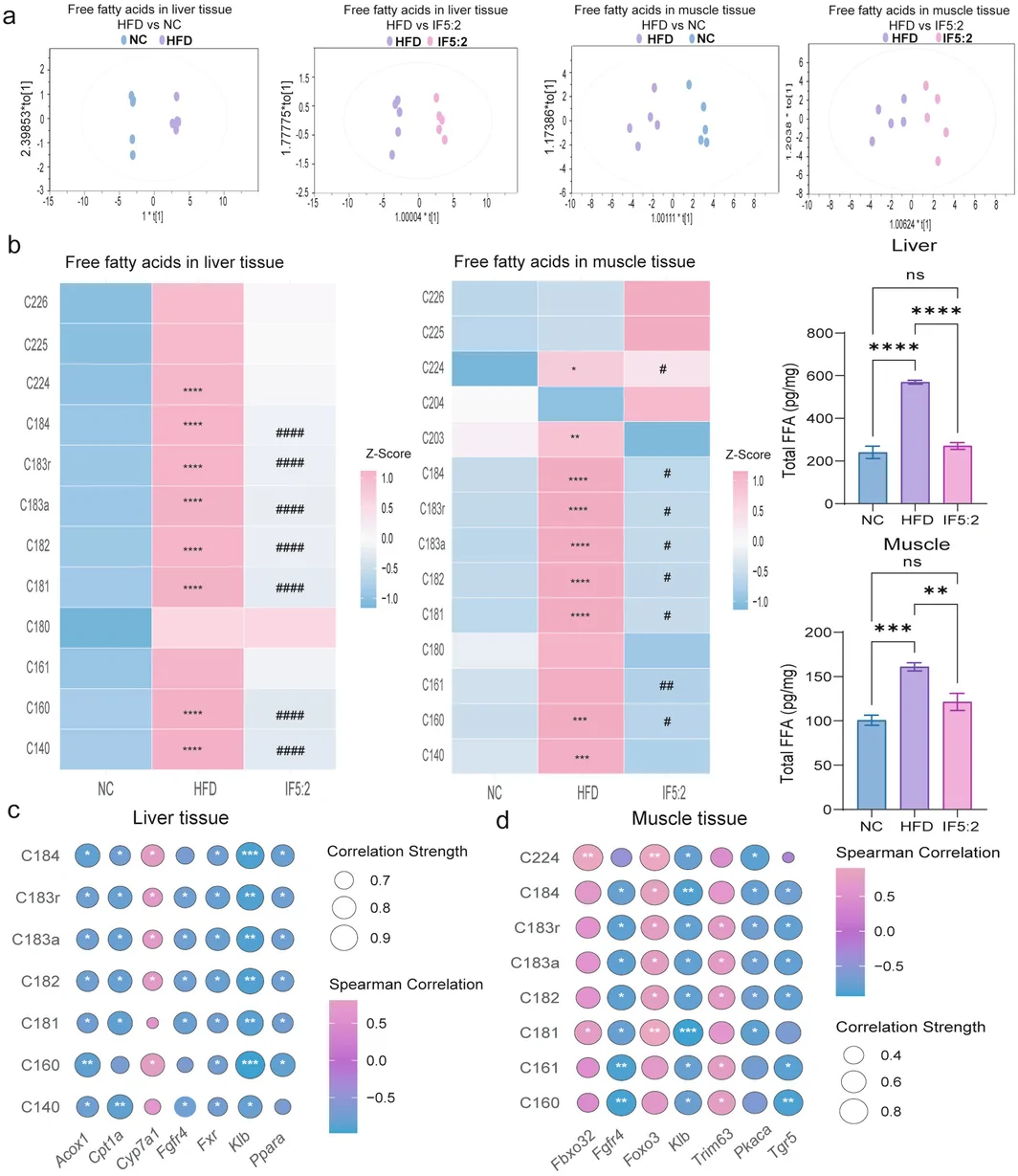
Free fatty acid profiles in liver and skeletal muscle, and correlation analysis of differential free fatty acids with bile acid–related genes. (a) OPLS‐DA analysis of free fatty acids in liver and skeletal muscle, with comparisons of HFD versus NC and HFD versus IF5:2. (b) Total free fatty acid contents and heatmap of individual free fatty acid levels in liver and skeletal muscle (*n* = 5). (c) Heatmap showing correlations between differential free fatty acids (HFD vs. IF5:2) in liver and bile acid receptor–related genes. (d) Heatmap showing correlations between differential free fatty acids (HFD vs. IF5:2) in skeletal muscle, bile acid receptor–related genes, and classic skeletal muscle atrophy–related genes. Larger circles in the heatmap indicate stronger correlation. A correlation coefficient > 0 denotes positive correlation, whereas < 0 denotes negative correlation. * indicates comparisons with the NC group, and # indicates comparisons with the HFD group. *, #, *p* < 0.05; **, ##, *p* < 0.01; ***, ###, *p* < 0.001; ****, ####, *p* < 0.0001.

### 3.7 Correlation Analysis Between Bile Acid Receptors/Downstream Genes and FFA Profiles in the Liver and Skeletal Muscle

Given the close relationship between bile acid signalling activation and lipid metabolism in the liver and muscle [17], Spearman correlation analysis was performed between differential FFAs and bile acid–responsive receptors and their downstream genes in the liver and skeletal muscle of IF5:2 and HFD mice. In the liver, expression of the canonical bile acid receptor *Fxr* and its downstream target genes was generally significantly negatively correlated with differential FFAs. *Klb* and *Fgfr4*, key receptors for FGF15, also showed significant negative correlations with multiple differential FFAs (Figure 6c). Notably, *Cyp7a1* was positively correlated with differential FFAs. Hepatic lipid accumulation suppresses FXR signalling. As a canonical negative‐feedback target of *Fxr*, *Cyp7a1* is transcriptionally repressed by the FXR‐SHP axis; reduced FXR activity alleviates this repression [18, 19], which may explain the positive association between FFAs and *Cyp7a1* expression. In skeletal muscle, the most significant negative correlation was observed between *Tgr5* and C16:0 (Figure 6d). Increased *Foxo3* (a downstream target of *Tgr5*) promotes expression of the muscle atrophy genes *Atrogin‐1* and *MuRF1* [20]. In our study, *Foxo3* and the two atrophy genes were generally positively correlated with FFAs, whereas *Pkaca* (another downstream target of *Tgr5*) was negatively correlated with FFAs (Figure 6d). These findings further support a potential mechanism whereby IF5:2 ameliorates MASLD and protects skeletal muscle from impairment, possibly by remodelling gut microbiota dysbiosis and modulating bile acid receptor signalling and fatty acid metabolism.

## 4 Discussion

Recent clinical studies on the IF5:2 regimen have demonstrated that IF5:2 is associated with greater reductions in fat mass and glycated haemoglobin in patients with MASLD, with no significant differences in body weight, lean body mass, insulin resistance, triglycerides or liver stiffness compared with continuous energy restriction [6]. In addition, IF5:2 effectively attenuates the progression of Western diet‐induced steatohepatitis [5]. Consistent with these prior reports, our work supports a protective role of IF5:2 against HFD‐driven MASLD and MASH progression. Uncontrolled MASLD leads to progressive hepatic lipid accumulation and exacerbated inflammation, which significantly increase the risk of skeletal muscle impairment [2]. As the major organ responsible for glucose disposal, loss of skeletal muscle mass promotes insulin resistance, which in turn aggravates MASLD [21]. Our mouse study likewise confirmed that prolonged HFD feeding exacerbated impairments in both the liver and skeletal muscle; however, IF5:2 intervention under the high‐fat background ameliorated MASLD while concurrently preventing the muscle impairment. Previous studies suggest that IF enhances metabolic flexibility by imposing a metabolic switch between fasting and feeding states, forcing the body to shift fuel sources and promoting catabolism of stored lipids in the liver and muscle [22]. The improvement in lipid handling and inflammatory status in liver and skeletal muscle under IF5:2 may, at least partially, be attributed to such metabolic reprogramming. Nevertheless, the regulatory basis underlying liver–skeletal muscle metabolic crosstalk remains incompletely defined.

Intestinal barrier integrity and microbiota‐derived metabolites are considered key mediators linking the gut, liver and skeletal muscle. In our study, IF5:2 improved intestinal barrier–related features and reshaped the gut microbial community, including enrichment of taxa previously associated with mucosal homeostasis, such as *Faecalibaculum rodentium*. Previous studies have associated this taxon with butyrate production and the maintenance of tight junction proteins, including ZO‐1 and Occludin, suggesting a potential role in mucosal barrier homeostasis [23]. By maintaining gut barrier integrity, IF5:2 may reduce microbial LPS leakage into the circulation. Under a HFD background, circulating LPS can induce systemic inflammation, hepatic injury and skeletal muscle dysfunction, making this effect biologically relevant [24, 25, 26]. In addition, accumulating evidence indicates that IF can modulate gut microbiota composition and improve intestinal barrier function [27, 28]. Collectively, these findings suggest that the protective effect of IF5:2 on the gut barrier may represent an initiating step in the improvement of hepatic and skeletal muscle metabolic abnormalities.

Beyond its barrier‐protective effects, our results suggest that IF5:2 may also regulate host metabolism through potential microbiota‐mediated changes in bile acid metabolism. Among the top three high‐scoring signature species in the IF5:2 group, *Limosilactobacillus reuteri* and *Limosilactobacillus* sp. have been confirmed to possess BSH activity [29]. Although direct evidence of BSH enzymatic activity in *F. rodentium* is lacking, existing studies have demonstrated that this strain is involved in the conversion of primary bile acids to SBAs [30]. Furthermore, supplementation with BSH‐active probiotics has been shown to ameliorate dyslipidaemia and alleviate hepatic steatosis and inflammation, effects that are closely associated with intestinal bile acid metabolism [31]. As key ligands for bile acid receptors, selected bile acids can activate intestinal FXR signalling and induce FGF15 expression [32]. The secreted FGF15 then acts on hepatic FGFR4 to suppress *Cyp7a1*, whereas hepatic FXR‐SHP signalling further inhibits bile acid synthesis and alleviates bile acid–induced lipotoxicity [18]. Meanwhile, FXR signalling has also been reported to modulate *Ppara* and *Cpt1a* expression, thereby promoting fatty acid β‐oxidation and reducing hepatic lipid accumulation [18, 19]. Previous mammalian studies suggest that increased SBA biosynthesis can reduce HFD‐induced lipid deposition [33]; moreover, as important microbiota‐derived metabolites, SBAs also regulate systemic immunity and energy metabolism in muscle and adipose tissue [34]. Therefore, the observed changes in bile acid receptor–related gene expression are consistent with the possibility that IF5:2 improves MASLD, at least in part, through modulation of bile acid signalling.

Importantly, bile acid signalling may also contribute to the improvement of skeletal muscle phenotype [35]. Previous mouse studies suggest that intestinal FXR–FGF15 signalling may contribute to skeletal muscle maintenance. Circulating FGF15 may potentially influence skeletal muscle through FGFR4/KLB signalling and attenuate the expression of muscle atrophy–related genes, including MuRF1 and Atrogin‐1; however, this endocrine mechanism was not directly established in the present study [36]. In addition, TGR5, a bile acid–activated G protein–coupled receptor in skeletal muscle, has been reported to improve muscle metabolism and function upon activation by specific agonists [37]. In the present study, IF5:2 upregulated the expression of *Tgr5* and *Pkaca*‐related genes in skeletal muscle, and their expression was negatively correlated with palmitic acid levels, with palmitic acid being a saturated FFA known to induce tissue inflammation [38]. This finding suggests that bile acid receptor signalling may be involved in attenuating palmitic acid–related lipotoxic stress. Thus, these results support the notion that bile acid receptor signalling may contribute to reduced muscle lipid deposition and protection against skeletal muscle impairment. Nevertheless, marked differences exist between humans and mice in terms of gut microbiota composition, BSH‐producing taxa and the enzymatic properties of BSH. Animal experiments typically employ standardised HFDs with short‐term interventions, whereas human studies are complicated by high heterogeneity in dietary habits, baseline metabolic status and gut microecology. The net effect of BSH activity on hepatic metabolism in human MASLD is state‐dependent: Elevated BSH activity may either mediate protective bile acid signalling or generate lipotoxic SBAs, depending on the specific metabolic context [39]. Therefore, additional human studies are required to determine whether microbiota‐mediated enhancement of BSH activity under IF5:2 fasting confers benefits to hepatic and skeletal muscle metabolic health. Although other mechanisms, such as liver‐derived exosomal communication, may also mediate gut‐liver‐muscle crosstalk [40], our data preliminarily suggest that IF5:2 exerts coordinated protective effects on the liver and skeletal muscle through microbiota‐dependent regulation of barrier function and bile acid metabolism.

Collectively, our findings support a putative gut–liver–muscle mechanism by which IF5:2 improves HFD‐induced MASLD and skeletal muscle impairment. Specifically, IF5:2 remodels gut microbiota and improves intestinal barrier‐related features. Metagenomic functional prediction suggested an increased potential for microbiota‐mediated SBA transformation, although this functional inference requires direct validation. Combined with the altered expression of bile acid receptors and downstream metabolic genes, these results are consistent with a putative regulatory pathway in which microbiota‐related bile acid signalling may contribute to the improved lipid metabolism and muscle homeostasis after IF5:2 intervention. The present study highlights the dual protective effects of IF5:2 on MASLD and skeletal muscle, providing a rationale for further evaluating IF5:2 as a potential nutritional intervention.

## 5 Limitations and Perspectives

Although our study demonstrates the dual beneficial effects of the IF5:2 regimen on both MASLD and skeletal muscle impairment and preliminarily elucidates the underlying mechanisms by which it ameliorates HFD‐induced MASLD and preserves skeletal muscle impairment, several limitations related to experimental design and clinical translation should be acknowledged. First, the present study was conducted in a diet‐induced MASLD mouse model. Although this model is widely used in metabolic liver disease research, it does not fully recapitulate the heterogeneity of human MASLD, which is influenced by age, sex, genetic background and long‐term dietary patterns. Moreover, marked species differences exist between rodents and humans with respect to bile acid composition, metabolic rate, feeding behaviour and gut microbiota structure. Therefore, the enrichment of BSH‐active bacteria and the downstream activation of bile acid receptor signalling observed in this study require further validation in human subjects. Second, from the perspective of experimental design, only male mice were used in the present study. Given that sex can markedly modulate the regulatory effects of IF5:2 on the liver and skeletal muscle, future studies are required to verify whether this intervention confers similar protective effects in female mice. Third, the mechanistic evidence obtained in this study remains largely correlative. Future studies combining faecal microbiota transplantation, antibiotic‐mediated gut microbiota depletion and mono‐colonisation with IF5:2‐enriched signature bacteria are warranted to clarify whether microbial alterations are necessary and sufficient for the observed metabolic benefits. Functional validation using FXR‐ or TGR5‐deficient mice, along with receptor‐specific agonists or antagonists, will also help define the causal contributions of these pathways. Finally, the clinical application of the IF5:2 regimen in patients with MASLD requires careful evaluation. Although IF represents a promising and feasible lifestyle intervention, its long‐term safety, adherence and efficacy vary depending on patients' metabolic backgrounds. In particular, MASLD patients who are elderly, diabetic, sarcopenic or at risk of insufficient protein intake may respond differently to fasting‐based interventions. Therefore, clinical trials should assess not only hepatic outcomes but also skeletal muscle quality, muscle strength, dietary intake, gut microbiota composition and bile acid profiles. Moreover, long‐term clinical trials in MASLD patients are needed to systematically evaluate the effects of IF5:2 on liver and skeletal muscle health, thereby confirming its translational value as an intervention strategy that ameliorates MASLD while protecting skeletal muscle against impairment.

## Author Contributions

RW designed the study. RW performed the experiments, analysed the data and wrote the manuscript. RY, DH, TX and ZY performed the experiments. RY and YG analysed the data. YN critically reviewed the manuscript. All authors read and approved the final manuscript.

## Funding

This study was supported by the Non‐communicable Chronic Diseases National Science and Technology Major Project, 2024ZD0531800, and the National Natural Science Foundation of China, 82473614.

## Ethics Statement

All animal procedures in this study were reviewed and approved by the Ethics Committee of Harbin Medical University and were performed in strict accordance with the ethical standards established in the 1964 Declaration of Helsinki and its subsequent amendments.

## Conflicts of Interest

The authors declare no conflicts of interest.

## Supporting information


**Table S1:** Diet composition.
**FIGURE S1:** Hepatic pathological and biochemical characteristics of the mouse MASD model established by a 14‐week 45% high‐fat diet intervention. (a) Representative images of haematoxylin and eosin (H&E) staining, F4/80 immunohistochemical staining and Oil Red O staining of mouse liver sections; (b) body weight (g) of mice; (c) quantitative analysis of the area fraction of lipid droplets in Oil Red O staining; (d) quantitative analysis of the percentage of F4/80‐positive area in the liver (%); (e–i) serum levels of triglycerides (TG), total cholesterol (TCHOL), glucose, alanine aminotransferase (ALT) and aspartate aminotransferase (AST). n = 5 mice per group. *p < 0.05, **p < 0.01, ***p < 0.001 and ****p < 0.0001.
**FIGURE S2:** Daily food intake and fat intake of mice in each group during the intervention period. (a) Mean daily total energy intake over 1 week (kcal). (b) Mean daily food intake over 1 week. (c) Mean daily fat‐derived energy intake over 1 week (kcal). (d) Mean daily fat intake over 1 week. (e) Cumulative weekly food intake. (f) Cumulative weekly fat intake. (g) Cumulative weekly fat‐derived energy intake (kcal). * indicates comparisons with the NC group, and # indicates comparisons with the HFD group. *, #, p < 0.05; **, ##, p < 0.01; ***, ###, p < 0.001; ****, ####, p < 0.0001.
**FIGURE S3:** Serum and hepatic inflammatory cytokine levels measured by ELISA after 12 weeks of the 5:2 intermittent fasting intervention. (a, b) Serum levels of interleukin‐6 (IL‐6) and tumour necrosis factor‐α (TNF‐α); (c, d) hepatic tissue levels of IL‐6 and TNF‐α. n = 5 mice per group. (e–j) Weights of subcutaneous, perirenal, epididymal, omental, brown and total white adipose tissue, respectively. *p < 0.05, **p < 0.01, ***p < 0.001 and ****p < 0.0001.
**FIGURE S4:** Absolute quadriceps mass, maximal grip strength and linear correlation analysis between quadriceps mass and body weight in mice. (a) Absolute quadriceps mass (g); (b) maximal grip strength (unadjusted for body weight); (c) linear correlation analysis between quadriceps mass and body weight. *p < 0.05 indicates statistically significant linear correlation. R2, coefficient of determination. n = 8 mice per group. *p < 0.05, **p < 0.01, ***p < 0.001 and **p < 0.0001; ns, not significant.
**FIGURE S5:** Gut microbiota richness, diversity and differential taxa following the 5:2 intermittent fasting (IF5) intervention. (a, b) Gut microbiota richness and diversity indices (Chao, Shannon, Ace and Simpson; higher values indicate greater richness and diversity). (e, f) Relative abundance comparison of the two most significantly differential taxa between the IF5 and HFD groups. n = 6 per group.
**FIGURE S6:** Permutation tests of OPLS‐DA models for liver and skeletal muscle tissue in mice. (a, b) Permutation tests for skeletal muscle tissue. (c, d) Permutation tests for liver tissue. Model validity and overfitting were assessed using permutation test parameters, with Q2 < 0.05 (typically negative) and R2 < 0.3–0.4 considered indicators of a well‐fitted model.

## Data Availability

The data that support the findings of this study are available from the corresponding author upon reasonable request.
