# Supplementary material for: A 5:2 Intermittent Fasting Regimen Ameliorates High‐Fat Diet‐Induced MASLD‐Associated Skeletal Muscle Impairment in Mice
Source: J Cachexia Sarcopenia Muscle. 2026 Oct 4;17(5):e70398. doi: 10.1002/jcsm.70398 (PMC13634929; doi:10.1002/jcsm.70398)
Supplement: Supplementary file 1 [file JCSM-17-e70398-s001.docx]

Supplementary Materials

**1. Materials and Methods**

Table S1: Diet Composition

|  | Normal diet (H10010) | | 45% High fat diet（H10045） | |
| --- | --- | --- | --- | --- |
| Component | g | Kcal | g | Kcal |
| Casein | 189.58 | 758.32 | 233.06 | 932.24 |
| Cystine | 2.84 | 11.36 | 3.5 | 14 |
| Corn Starch | 298.59 | 1194.36 | 84.83 | 339.32 |
| Maltodextrin | 33.18 | 132.72 | 116.53 | 466.12 |
| Sucrose | 331.77 | 1327.08 | 201.36 | 805.44 |
| Cellulose | 47.4 | 0 | 58.26 | 0 |
| Soybean Oil | 23.7 | 213.3 | 29.13 | 262.17 |
| Lard | 18.96 | 170.64 | 206.84 | 1861.56 |
| Mineral Mixture M1002 | 9.48 | 0 | 11.65 | 0 |
| Calcium Hydrogen Phosphate | 12.32 | 0 | 15.15 | 0 |
| Calcium Carbonate | 5.21 | 0 | 6.41 | 0 |
| Potassium Citrate | 15.64 | 0 | 19.23 | 0 |
| Vitamin Mixture V1001 | 9.48 | 37.92 | 11.56 | 46.24 |
| Choline Bitartrate | 1.9 | 0 | 2.33 | 0 |
| Food Colorant | 0.047 | 0 | 0.058 | 0 |
|  | Mass ratio (g%) | Energy ratio (Kcal%) | Mass ratio (g%) | Energy ratio (Kcal%) |
| Protein | 19.2 | 20 | 24 | 20 |
| Carbohydrate | 67.3 | 70 | 41 | 35 |
| Fat | 4.3 | 10 | 24 | 45 |
| Kcal/g | 3.85 | | 4.73 | |

1.1 ***Hematoxylin and Eosin (H&E) Staining of Liver, Colon, and Skeletal Muscle***

***Tissues***

(1) *Paraffin-Embedded Tissue Sectioning Protocol*
1. **Tissue Collection and Fixation**: Fresh tissues were fixed in 4% paraformaldehyde

for more than 24 hours. The tissues were removed from the fixative, and the target regions were trimmed neatly with a scalpel in a fume hood. The trimmed tissues and corresponding labels were placed in dehydration cassettes.

2. **Dehydration**: The dehydration cassettes were placed in a basket and subjected to

gradient alcohol dehydration in a dehydrator in the following order: 75% alcohol for 4 h → 85% alcohol for 2 h → 90% alcohol for 2 h → 95% alcohol for 1 h → absolute ethanol I for 30 min → absolute ethanol II for 30 min → alcohol-benzene for 5–10 min → xylene I for 5–10 min → xylene II for 5–10 min → paraffin I for 1 h → paraffin II for 1 h → paraffin III for 1 h.

3. **Embedding**: The paraffin-impregnated tissues were embedded in an embedding

machine. Melted paraffin was first added to the embedding mold. Before the paraffin solidified, the tissues were taken out of the dehydration cassettes, placed in the embedding mold according to the requirements of the embedding surface, and labeled correspondingly. The mold was cooled on a -20°C freezing stage. After the paraffin solidified, the paraffin block was removed from the mold and trimmed.

4. **Sectioning**: The trimmed paraffin block was sectioned on a paraffin microtome at

a thickness of 4 μm. The sections were floated on the warm water (40°C) of a flotation bath to flatten the tissues, fished out with glass slides, and placed in a 60°C oven for baking. After the water was dried and the paraffin was melted, the slides were taken out and stored at room temperature for later use.

(2) *Hematoxylin and Eosin (H&E) Staining Protocol*
1. **Deparaffinization and Rehydration**: The paraffin sections were sequentially

immersed in xylene I for 20 min → xylene II for 20 min → absolute ethanol I for 10 min → absolute ethanol II for 10 min → 95% alcohol for 5 min → 90% alcohol for 5 min → 80% alcohol for 5 min → 70% alcohol for 5 min → rinsed with distilled water.

2. **Hematoxylin Staining for Nuclei**: The sections were stained with Harris

hematoxylin for 3–8 min, rinsed with tap water, differentiated with 1% hydrochloric acid alcohol for several seconds, rinsed with tap water, blued with 0.6% ammonia water, and washed with running water.

3. **Eosin Staining for Cytoplasm**: The sections were stained in eosin solution for 1-3

min.

4. **Dehydration and Mounting**: The sections were dehydrated and cleared by

sequential immersion in 95% alcohol I for 5 min → 95% alcohol II for 5 min → absolute ethanol I for 5 min → absolute ethanol II for 5 min → xylene I for 5 min → xylene II for 5 min. The sections were taken out of xylene, air-dried slightly, and mounted with neutral balsam.

5. **Microscopic Examination and Image Acquisition**: The sections were observed

under a microscope, and images were collected and analyzed.

1.2 ***Liver Immunohistochemical Staining Protocol (F4/80)***

1. **Deparaffinization and Rehydration**: The paraffin sections were sequentially

immersed in xylene I for 15 min → xylene II for 15 min → absolute ethanol I for 5 min → absolute ethanol II for 5 min → 85% alcohol for 5 min → 75% alcohol for 5 min → rinsed with distilled water.

2. **Antigen Retrieval**: The tissue sections were placed in a retrieval box filled with

citrate antigen retrieval buffer (pH 6.0) and subjected to antigen retrieval in a microwave oven. The citrate buffer (pH 6.0) was heated at high power for 5 min, turned off for 5 min, and this process was repeated 3 times. During this process, excessive evaporation of the buffer should be prevented, and the sections must not be dried. After natural cooling, the slides were placed in PBS (pH 7.4) and washed with shaking on a decolorizing shaker 3 times, 5 min each time.

3. **Blocking**: The sections were placed in 3% hydrogen peroxide solution, incubated

at room temperature in the dark for 25 min to block endogenous peroxidase. Then the slides were placed in PBS (pH 7.4) and washed with shaking on a decolorizing shaker 3 times, 5 min each time.

4. **Circling**: After the sections were slightly dried, a immunohistochemical pen was

used to draw a circle around the tissues (to prevent antibody runoff).

5. **Serum Blocking**: 3% BSA was added dropwise into the circle and incubated at roo

m temperature for 30 min.

6. **Primary Antibody Incubation**: The blocking solution was gently discarded, and

the primary antibody diluted with PBS at a certain ratio was added dropwise onto the sections. The sections were placed flat in a humid chamber and incubated at 4°C overnight (a small amount of water was added to the humid chamber to prevent antibody evaporation).

7. **Secondary Antibody Incubation**: The slides were placed in PBS (pH 7.4) and

washed with shaking on a decolorizing shaker 3 times, 5 min each time. After the sections were slightly dried, the secondary antibody corresponding to the species of the primary antibody was added dropwise into the circle to cover the tissues, and incubated at room temperature in the dark for 50 min.

8. **DAB Chromogenic Reaction and Nuclear Counterstaining**: The slides were

placed in PBS (pH 7.4) and washed with shaking on a decolorizing shaker 3 times, 5 min each time. After the sections were slightly dried, DAB chromogenic agent was added dropwise into the circle, and the chromogenic process was controlled under a microscope. After complete chromogenic reaction, the sections were rinsed with distilled water or tap water, counterstained with hematoxylin, differentiated with 1% hydrochloric acid alcohol (about 1 second), rinsed with tap water, blued with ammonia water, and washed with running water.

9. **Dehydration and Mounting**: The sections were dehydrated and cleared by

sequential immersion in 75% alcohol for 5 min → 85% alcohol for 5 min → absolute ethanol I for 5 min → absolute ethanol II for 5 min → xylene I for 5 min. The sections were taken out of xylene, air-dried slightly, and mounted with neutral balsam.

***1.3. Determination of Free Fatty Acids in Liver and Skeletal Muscle Tissues***

*(1) Homogenization and Extraction*

Approximately 50 mg of liver tissue and 100 mg of skeletal muscle tissue were weighed into 2 ml homogenization tubes, and 1 ml of PBS was added. After sufficient homogenization three times using a homogenizer, the homogenate was transferred to a 15 ml EP tube. 1.5 ml of isopropanol:n-hexane (3:2, v/v) was added, followed by vortexing for 1 minute and standing for extraction for 2 hours. Then, centrifugation was performed at 3500 rpm for 10 minutes. The centrifuged liquid was divided into three layers (the upper organic phase, the lower aqueous phase and tissue residues). The upper organic phase was collected into a new 15 ml EP tube. 1.5 ml of isopropanol:n-hexane (3:2, v/v) was added again to the remaining tissue residues and aqueous phase. After vortexing and centrifugation, the upper organic phase was collected again. The two extracted organic phases were mixed and dried under nitrogen.

(2) *Methyl Esterification*

2 ml of 0.4 M methanolic potassium hydroxide was added to the dried 1.5 ml EP tube, suspended for 1 minute, and placed at room temperature for 10 minutes. Then, the mixture was transferred to a 15 ml EP tube, an appropriate amount of anhydrous sodium sulfate was added, followed by 2 ml of n-hexane. After vortexing for 1 minute, centrifugation was performed. The supernatant was discarded, 2 ml of 10% methanolic sulfuric acid was added, suspended for 1 minute, and incubated in a 62°C water bath for 2 hours. The tube was taken out and cooled for 15 minutes. An appropriate amount of anhydrous sodium sulfate and 2 ml of n-hexane were added to the cooled tube, suspended for 1 minute, then centrifuged at 3500 rpm for 5 minutes. The supernatant was collected into a 1.5 ml EP tube and dried under nitrogen. The sample was dissolved in 100 μl of n-hexane, vortexed for 1 minute, and 60 μl was taken for GC-MS analysis.

2. **Result**

**Fig. S1** Hepatic pathological and biochemical characteristics of the mouse MASD model established by 14-week 45% high-fat diet intervention. (a) Representative images of hematoxylin and eosin (H&E) staining, F4/80 immunohistochemical staining, and Oil Red O staining of mouse liver sections; (b) body weight (g) of mice; (c) quantitative analysis of the area fraction of lipid droplets in Oil Red O staining; (d) quantitative analysis of the percentage of F4/80-positive area in the liver (%); (e–i) serum levels of triglycerides (TG), total cholesterol (TCHOL), glucose, alanine aminotransferase (ALT), and aspartate aminotransferase (AST). (n = 5 mice per group) * *p* < 0.05, ** *p* < 0.01, *** *p* < 0.001, **** *p* < 0.0001.

**Fig. S2** Daily food intake and fat intake of mice in each group during the intervention period. (a) Mean daily total energy intake over one week (kcal); (b) Mean daily food intake over one week; (c) Mean daily fat-derived energy intake over one week (kcal); (d) Mean daily fat intake over one week; (e) Cumulative weekly food intake; (f) Cumulative weekly fat intake; (g) Cumulative weekly fat-derived energy intake (kcal). * indicates comparisons with the NC group, and # indicates comparisons with the HFD group. *, #, *p* < 0.05; **, ##, *p* < 0.01; ***, ###, *p* < 0.001; ****, ####, *p* < 0.0001.

**Fig. S3** Serum and hepatic inflammatory cytokine levels measured by ELISA after 12 weeks of the 5:2 intermittent fasting intervention. (a, b) Serum levels of interleukin-6 (IL-6) and tumor necrosis factor-α (TNF-α); (c, d) hepatic tissue levels of IL-6 and TNF-α. n = 5 mice per group. (e–j) Weights of subcutaneous, perirenal, epididymal, omental, brown, and total white adipose tissue, respectively. * *p* < 0.05, ** *p* < 0.01, *** *p* < 0.001, **** *p* < 0.0001.

**Fig. S4** Absolute quadriceps mass, maximal grip strength, and linear correlation analysis between quadriceps mass and body weight in mice. (a) Absolute quadriceps mass (g); (b) maximal grip strength (unadjusted for body weight); (c) linear correlation analysis between quadriceps mass and body weight. *p < 0.05 indicates statistically significant linear correlation. R², coefficient of determination. n = 8 mice per group. *p < 0.05, **p < 0.01, ***p < 0.001, **p < 0.0001; ns, not significant.

**Fig. S5** Gut microbiota richness, diversity, and differential taxa following the 5:2 intermittent fasting (IF5) intervention. (a, b) Gut microbiota richness and diversity indices (Chao, Shannon, Ace, and Simpson; higher values indicate greater richness and diversity). (e, f) Relative abundance comparison of the two most significantly differential taxa between the IF5 and HFD groups. n = 6 per group.

**Fig. S6** Permutation tests of OPLS-DA models for liver and skeletal muscle tissue in mice. (a, b) Permutation tests for skeletal muscle tissue; (c, d) Permutation tests for liver tissue. Model validity and overfitting were assessed using permutation test parameters, with Q2 < 0.05 (typically negative) and R2 < 0.3–0.4 considered indicators of a well-fitted model.
